# Medical alliances and diabetes-related distress in China: role of self-efficacy as a partial mediator

**DOI:** 10.1093/inthealth/ihaf040

**Published:** 2025-04-16

**Authors:** Chenyu Zhou, Elizabeth Maitland, Stephen Nicholas, Xiaoyu Tian, Rugang Liu

**Affiliations:** School of Health Policy & Management, Nanjing Medical University, Nanjing 211166, China; Laboratory for Digital Intelligence & Health Governance, Nanjing Medical University, Nanjing 211166, China; Jiangsu Provincial Institute of Health, Nanjing Medical University, Nanjing 211166, China; Center for Global Health, Nanjing Medical University, Nanjing 211166, China; School of Management, University of Liverpool, Liverpool L697ZH, UK; Health Services Research and Workforce Innovation Centre, Newcastle Business School, University of Newcastle, Newcastle, NSW, Australia; Australian National Institute of Management and Commerce, NSW, Australia; School of Health Policy & Management, Nanjing Medical University, Nanjing 211166, China; School of Health Policy & Management, Nanjing Medical University, Nanjing 211166, China; Laboratory for Digital Intelligence & Health Governance, Nanjing Medical University, Nanjing 211166, China; Jiangsu Provincial Institute of Health, Nanjing Medical University, Nanjing 211166, China; Center for Global Health, Nanjing Medical University, Nanjing 211166, China

**Keywords:** compact medical alliance, determinants, diabetes-related distress, mediator, self-efficacy

## Abstract

**Background:**

The prevalence of diabetes-related distress is high among diabetes mellitus patients, causing physical, psychological and economic burdens. China's general medical alliances and compact general alliances provide treatment for patients with type 2 diabetes mellitus (T2DM). This study aims to analyse the influence of medical alliances on diabetes-related distress and verified the mediating role of self-efficacy among T2DM patients.

**Methods:**

From one general and one compact medical alliance, data on 2156 T2DM adults >45 y of age were investigated through a questionnaire survey conducted in China. Diabetes-related distress, medical alliance data, self-efficacy information and control variables were collected. Hierarchical linear regression mediation analysis was used to analyse the influence of compact medical alliances and general medical alliances on diabetes-related distress and to verify self-efficacy as a mediator between medical alliances and diabetes-related distress.

**Results:**

Most patients (94.57%) with T2DM were suffering from diabetes-related distress, with an average score of diabetes-related distress (11.77±7.65). The respondents from compact medical alliances had lower diabetes-related distress (11.08±8.64) than from general medical alliances (12.38±6.61). Self-efficacy mediated the association between the type of medical alliance and diabetes-related distress (p<0.05). Higher income, lower health expenditure, lower education level, less sleep time, low physical exercise and low diabetes cognition were significant risk factors of diabetes-related distress (p<0.05).

**Conclusions:**

Compact medical alliances reduced diabetic patients’ diabetes-related distress significantly more than general medical alliances. Self-efficacy was a mediator between medical alliances and diabetes-related distress. Accelerating the transformation of the compact medical alliances can decrease diabetes-related distress and provide an integrated program of education, diabetes cognition and optimal sleep and exercise regimens to reduce diabetes-related distress.

## Introduction

Diabetes mellitus (DM) is a global public health threat. According to the Diabetes Atlas of the International Diabetes Federation (IDF), the number of adults with diabetes was 537 million worldwide in 2021 and was estimated to reach 783 million by 2045, with one in eight adults diagnosed with diabetes. China has the largest number of people with diabetes, reaching 50 million in 2021 and estimated to increase to 174.4 million by 2045. Living with DM is both challenging and stressful, with diabetes imposing physical, psychological and economic burdens on diabetes sufferers.^1,2^ Patients with diabetes have a higher risk of mental problems than non-diabetes sufferers,^3,4^ with diabetes-related distress a special psychological problem,^5,6^ affecting 22–36% of adults with DM.^7,8^ Diabetes-related distress triggers a series of negative consequences: reduced patient adherence to treatment and control of diabetes; not following medical advice on medication or healthy life activities; poor blood glucose control;^9–13^ degradation of quality of life; productivity loss; adverse health outcomes, such as pregnancy failures; and risk of excess mortality.^14–17^ One of the main goals of health management among diabetes patients is glycaemic control. A previous study conducted among 254 Chinese diabetes patients found that depression influenced glycaemic control through diabetes self-management and another study found that diabetes emotional distress mediated the association between depressive symptoms and glycaemic control.^18^

The influencing factors, and their action mechanisms, on diabetes-related distress is a key DM research area. A major area underpinning experiences of diabetes distress is not feeling in control.^19^ Previous studies have analysed the influencing factors of diabetes-related distress, with higher self-efficacy,^20,21^ longer sleep time,^21^ physical activities^22^ and diabetes cognition^23^ being significantly correlated with lower diabetes-related distress, while lower education levels,^24–26^ smoking and alcohol consumption status^25^ and sedentary lifestyle^27^ were risk factor positively correlated with higher diabetes-related distress. As a motivational factor, self-efficacy is a critical concept in social cognitive theory, related to one's belief and confidence in the ability to successfully perform tasks and execute skills effectively.^28^ A high degree of self-efficacy is essential for carrying out self-care diabetes behaviours, including directly improving diabetes self-management, medication adherence, metabolic outcomes and health-related quality of life.^29,30^ Self-efficacy has been frequently used as a mediating variable in diabetic population studies. A study in Hainan province in China found that diabetes stigma and self-efficacy exerted a chain mediation effect on the association between social support and diabetes distress.^31^ A study of 265 Chinese diabetes sufferers found that the association between knowledge and diabetes self-management behaviours was partially mediated by self-efficacy.^32^ A survey of adults with type 2 diabetes mellitus (T2DM) from 31 rural clinics in China found that self-efficacy mediated the relationships between both diabetes distress and depressive symptoms and self-care behaviours, glycaemic control and health-related quality of life.^33^ A study of 254 outpatients from a Beijing hospital found that depression and diabetes distress had only indirect effects on glycaemic control through both diabetes self-efficacy and diabetes self-management.^18^ A study conducted in the south of Iran revealed that diabetes management self-efficacy fully mediated the correlation between diabetes distress and resilience.^34^

Diabetes management also depends on the types of public health services available to diabetes sufferers. Medical alliances emerged out of China's 2009 health reforms, addressing the resource imbalances in China's tier hospital system, comprising primary urban and rural community health centres, secondary hospitals and tertiary hospitals.^35^ Promoting integrated medical treatment by combining the technical and professional resources of primary, secondary and tertiary hospitals, two types of medical alliances emerged. In 2016, general medical alliances were piloted and compact medical alliances were piloted in 2019. In general medical alliances, the separate tiers of the medical system cooperated in providing medical treatment but maintained their autonomy and independence in operation, finance and management. Compact alliances created a platform to control personnel and finances and manage the facilities in the multitier primary, secondary and tertiary hospital alliance, optimizing the integration of internal resources.^36^ In compact medical alliances, diabetes patients can access immediate healthcare and follow-up service to monitor their health status and provide timely interventions, such as blood glucose monitoring, inquiry about diabetes symptoms, medication guidance and health behaviour guidance.

While previous studies have developed a multi-attribute group decision-making evaluation framework to evaluate medical alliances and analysed the effect of medical alliances on health service utilization and the control of medical costs,^35,37^ there has been no study analysing the effects of general and compact medical alliances on diabetes-related distress. In this study, we analysed the influence of medical alliances on diabetes-related distress and verified the mediating role of self-efficacy among T2DM patients.

## Methods

### Sampling method

A questionnaire survey was conducted in Binhai, Jiangsu province, in May 2022, where the compact medical alliance pilot scheme was well established. The inclusion criteria were adults patients with T2DM and the exclusion criterion was patients who have other serious metabolic diseases. According to the sample size calculation formula, the sample size should be ≥864. We collected self-reported data on 1011 patients in compact medical alliances and 1145 patients in general medical alliances with an effective response rate was 98.31%.

### Mediator hypothesis

**Figure 1.**
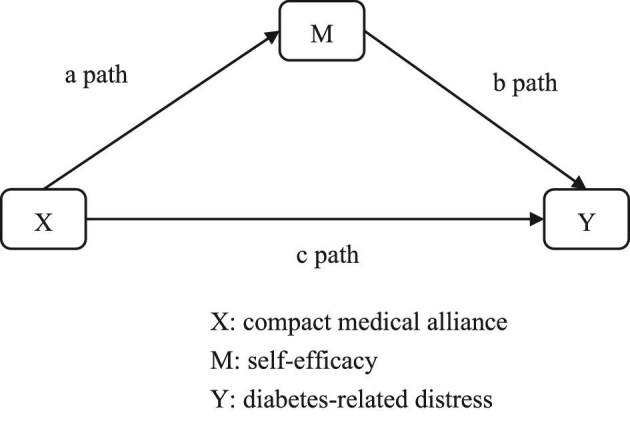
Mediator model.

Figure 1 shows the traditional hierarchical linear regression mediation analysis^38^ where the *c* path is the direct compact medical alliance–diabetes-related stress relationship, the *a* path represents the compact medical alliance and self-efficacy mediator effect, the *b* path represents the self-efficacy mediator–diabetes-related stress effect and the *c*′ path represents the direct compact medical alliance–diabetes-related stress in the following three equations:


(1) \begin{eqnarray*}
{\mathrm{Y}} = {{\mathrm{i}}_1} + {\mathrm{cX}} + {{\mathrm{d}}_1}{\mathrm{Z}} + {\varepsilon _1}\end{eqnarray*}

(2) \begin{eqnarray*}
{\mathrm{M}} = {{\mathrm{i}}_2} + {\mathrm{aX}} + {{\mathrm{d}}_2}{\mathrm{Z}} + {\varepsilon _2}\end{eqnarray*}

(3) \begin{eqnarray*}
{\mathrm{Y}} = {{\mathrm{i}}_3} + {\mathrm{c^{\prime}X}} + {\mathrm{bM}} + {{\mathrm{d}}_3}{\mathrm{Z}} + {\varepsilon _3}\end{eqnarray*}

where Z represents a set of confounders. When the compact medical alliance no longer influences diabetes-related stress after the self-efficacy mediator has been controlled there is complete mediation and when the compact medical alliance's influence on the diabetes-related stress is reduced after the self-efficacy mediator is controlled, then there is partial mediation of the relationship.

### Measurement of variables

#### Measurement of diabetes-related distress

The problem areas in diabetes (PAID) scale is widely used to measure diabetes-related distress and shows good psychometric validity compared with alternative measures.^39,40^ Twenty items with Likert scale answers ranging from 0 to 4 were scored by respondents. The total score ranged from 0 to 80, with higher scores indicating higher psychological pressure and more severe psychological distress.

#### Measurement of medical alliances

The respondents came from two types of medical alliances: compact and general.

#### Measurement of self-efficacy

Self-efficacy was measured using the widely accepted Self-efficacy for Managing Chronic Disease Six-Item Scale (SECD6).^41^ Six items assessed respondents’ confidence in their ability to take action, with a total score ranging from 0 to 60. Higher scores indicate higher self-efficacy.

#### Measurement of control variables

As shown in Table 1, the control variables included sex, age, average income per year, education level, marriage status, occupation status, medical insurance, health expenditures, sleep time, physical exercise, number of children, self-rated health and diabetes cognition. Respondents were divided into five age groups; three income groups (low, medium and high); employed, unemployed and retired occupational status; basic medical insurance for urban and rural residents (BMIURR), basic medical insurance for urban workers (BMIUW), without medical insurance and other groups. Health expenditures were measured by health expenditures in the past year and were divided into low, medium and high groups. Sleep time was divided into three groups and self-rated health ranged from 0 to 100, with 0 as death and 100 as complete health. The Audit of Diabetes Knowledge was used to measure diabetes cognition or patients’ awareness and understanding of diabetes and diabetes management information.^42^ The items were scored know/not know, comprising questions on diet, treatment, disease, foot care, the influence of physical exercise, the influence of smoking and drinking, the risk of complications and hypoglycaemia.

**Table 1.** The characteristics of respondents (N=2156)

Categorical variables		n	%
Medical alliance	General	1145	53.11
	Compact	1011	46.89
Sex	Male	785	36.41
	Female	1371	63.59
Age (years)	<60	389	18.04
	≥60–<65	323	14.98
	≥65–<70	480	22.26
	≥70–<75	503	23.33
	≥75	461	21.38
Income (RMB)	Low (<2000)	257	11.92
	Medium (≥2000–<4000)	1136	52.69
	High (≥4000)	763	35.39
Education	Primary school and below	1612	74.77
	Middle school	377	17.49
	High school and above	167	7.75
Marriage	Married cohabitation	1686	78.20
	Married and separated	111	5.15
	Divorced	21	0.97
	Widow	317	14.70
	Single	21	0.97
Occupation status	Employed	732	33.95
	Unemployed	1370	63.54
	Retired	54	2.50
Medical insurance	BMIURR	1988	92.21
	Without medical insurance	15	0.70
	BMIUW	19	0.88
	Others	134	6.22
Health expenditures (RMB)	Low (<2000)	715	33.16
	Medium (≥2000–<3000)	539	25.00
	High (≥3000)	902	41.84
Sleep time (hours)	<6	641	29.73
	6–8	1394	64.66
	>8	121	5.61
Physical exercise	No	324	15.03
	Yes	1832	84.97
Continuous variables		Mean	SD
Number of children		2.48	1.00
Self-rated health		72.83	12.05
Self-efficacy		38.36	7.72
Diabetes cognition		51.89	16.61
Diabetes-related distress		11.77	7.65

### Statistical methods

Mean and standard deviation (SD) were used to describe the continuous variables and the percentage ratio was used to describe categorical variables. Analysis of variance (ANOVA) was used to analyse the differences in diabetes-related distress by categorical variables. Pearson correlation analysis was used to test the correlation between diabetes-related distress and the continuous variables and liner regression was used to analyse the determinants of diabetes-related distress and to verify the mediator. The significance level was p<0.05.

## Results

### Characteristics of respondents

Table 1 shows the respondents’ characteristics: the average age was 67.73±8.31 y, 63.59% were female, the average income was RMB 5090.68±10 940.02, 74.77% had a primary school or lower education level with only 7.75% educated at high school and above, 78.2% were married or living together, 33.95% were employed and 63.54% were unemployed and 92.21% had basic medical insurance for urban and rural residents. From Table 1, 29.73% of respondents slept <6 h/d and 64.66% slept 6–8 h/d, 84.97% had regular physical exercise, they had an average of 2.48±1 children, the self-rated health average score was 72.83±12.05 (on a 10–100 scale), the self-efficacy average score was 38.36±7.72 (on a 10–60 scale), the diabetes cognition average score was 51.89±16.61 (on a 10–92 scale) and the average health expenditure per year was RMB9022.67±334 312.37. The number of respondents in both types of medical alliances was roughly equal, with 46.89% in compact alliances and 53.11% in general alliances. The average diabetes-related distress score was 11.77±7.65 (on a 0–80 scale), with only 5.43% of respondents reporting no diabetes-related distress.

### The results of single factor analysis

Table 2 shows the results of the single factor analysis. For the independent variables, the differences in diabetes-related distress among subgroups were tested by ANOVA. There were significant differences in diabetes-related distress by sex, income, education, health expenditures, sleep time, physical exercise and medical alliance group (p<0.05). Age, marriage status, occupation and medical insurance did not show any significant influence on diabetes-related distress (p>0.05). The diabetes-related distress score of respondents in a compact medical alliance (11.08) was significantly lower than that of respondents in a general medical alliance (12.38; p<0.001). The diabetes-related distress score of females (11.25) was higher than that of males (11.12; p=0.03); both medium- (12.04) and high-income (11.79) groups had higher diabetes-related distress scores than the low-income group (10.52; p=0.016); the diabetes-related distress score decreased as the education level increased (p<0.001), with respondents with high school and above scoring lowest (10.15) and the primary school and below group scoring highest (12.24). Diabetes-related distress also decreased with health expenditures and when sleep time increased (p<0.001). Respondents who participated regularly in physical exercise scored significantly lower (11.3) than respondents without regular exercise (14.43; p<0.001).

**Table 2.** The results of single factor analysis.

Categorical variables		Mean	SD	F	p-Value
Sex	Male	11.12	7.49	9.16	0.003
	Female	12.15	7.73		
Age (years)	<60	11.21	7.58	0.68	0.602
	≥60–<65	11.76	7.34		
	≥65–<70	11.97	8.04		
	≥70–<75	11.95	7.46		
	≥75	11.87	7.74		
Income (RMB)	Low (<2000)	10.52	7.47	4.16	0.016
	Medium (≥2000–<4000)	12.04	8.12		
	High (≥4000)	11.79	6.93		
Education	Primary school and below	12.24	7.76	11.87	<0.001
	Middle school	10.5	7.07		
	High school and above	10.15	7.43		
Marriage	Married cohabitation	11.82	7.59	1.48	0.207
	Married and separated	11.24	8.28		
	Divorced	15.38	7.68		
	Widow	11.57	7.78		
	Single	10.67	7.19		
Occupation status	Employed	12.30	6.69	2.73	0.065
	Unemployed	11.48	8.02		
	Retired	11.98	9.90		
Medical insurance	BMIURR	11.72	7.55	0.42	0.739
	Without medical insurance	12.33	9.32		
	BMIUW	12.42	10.21		
	Others	12.42	8.57		
Health expenditures (RMB)	Low (<2000)	13.14	7.88	17.26	<0.001
	Medium (≥2000–<3000)	11.02	7.06		
	High (≥3000)	11.14	7.68		
Sleep time (hours)	<6	12.94	7.81	27.72	<0.001
	6–8	11.61	7.40		
	>8	7.45	8.07		
Physical exercise	Yes	14.43	8.83	46.96	<0.001
	No	11.30	7.33		
Medical alliance	Compact	11.08	8.64	15.62	<0.001
	General	12.38	6.61		
Continuous variables		Pearson correlation coefficient			p-Value
Number of children		−0.0006			0.9769
Self-rated health		−0.0277			0.1981
Self-efficacy		−0.3249			<0.001
Diabetes cognition		−0.1773			<0.001

Pearson correlation analysis was used to test the correlation between diabetes-related distress and the continuous independent variables. The results in Table 2 indicate that there were significant correlations between diabetes-related distress and self-efficacy and diabetes cognition (p<0.001), but no significant correlations between diabetes-related distress and self-rated health and number of children (p>0.05).

### The results of the regressions

As shown in Table 3, models 1, 2 and 3 tested the self-efficacy mediation hypothesis set out in equations 1–3 above. The dependent variable in models 1 and 3 was diabetes-related distress, while in model 2, self-efficacy for managing chronic diabetes was the dependent variable. The type of medical alliance and those control variables that were significant in univariate analyses were included in the models.

**Table 3.** The results of multiple liner regression models.

		Model 1		Model 2		Model 3	
Variables		Coef.	p-Value	Coef.	p-Value	Coef.	p-Value
Medical alliance	General	Reference group					
	Compact	−1.537	<0.001	2.673	<0.001	−0.750	0.017
Self-efficacy						−0.294	<0.001
Control variables							
Sex	Male	Reference group					
	Female	0.533	0.144	−1.281	0.001	0.156	0.655
Income (RMB)	Low (<2000)	Reference group					
	Medium (≥2000–<4000)	1.263	0.013	−0.935	0.071	0.987	0.042
	High (≥4000)	1.825	0.001	−0.475	0.382	1.686	0.001
Education	Primary school and below	Reference group					
	Middle school	−1.383	0.002	0.208	0.651	−1.322	0.002
	High school and above	−1.702	0.007	1.758	0.006	−1.184	0.048
Health expenditures (RMB)	Low (<2000)	Reference group					
	Medium (≥2000–<3000)	−2.473	<0.001	−0.714	0.094	−2.683	<0.001
	High (≥3000)	−2.573	<0.001	−0.707	0.060	−2.781	<0.001
Sleep time (hours)	<6	Reference group					
	6–8	0.092	0.805	0.406	0.287	0.212	0.553
	>8	−4.222	<0.001	0.098	0.895	−4.193	<0.001
Physical exercise	No	Reference group					
	Yes	−2.058	<0.001	3.017	<0.001	−1.170	0.009
Diabetes cognition		−0.084	<0.001	0.028	0.010	−0.076	<0.001

Coef.: coefficient.

The value of coefficient *c* is −1.537 with p<0.001 in model 1. It indicates that the type of medical alliance had a statistically significant impact on diabetes-related distress. Diabetic patients in compact medical alliances exhibit significantly lower levels of diabetes-related distress compared with those in general medical alliances. The *a* coefficient (2.673; p<0.001) in model 2 shows that the type of medical alliance significantly impacted the self-efficacy for managing diabetes. Diabetic patients in compact medical alliances had significantly higher self-efficacy for managing chronic disease compared with those in general medical alliances. The value of coefficient *c*′ was −0.75 (p=0.017) and the value of coefficient *b* was −0.294 (p<0.001) in model 3, which indicates that when both self-efficacy and the type of medical alliance were included in the model, the type of medical alliance continued to significantly influence diabetes-related distress, but self-efficacy partially mediated the type of medical alliance and distress relationship. Higher self-efficacy for managing chronic disease in diabetic patients was associated with lower levels of diabetes-related distress.

For the control variables, income, education, health expenditure, sleep time, physical exercise and diabetes cognition had a significant influence on diabetes-related distress (p<0.05). The diabetes-related distress level increased when income increased (β>0, p<0.05) and decreased with education level and higher health expenditures (β<0, p>0.05). The diabetes-related distress level of respondents who sleep >8 h/d was lower than respondents with <6 h of sleep per day (β=−4.193, p<0.001). The diabetes-related distress of respondents who participated in physical exercise regularly was lower than that of respondents without exercise (β=−1.17, p=0.009) and diabetes cognition had a negative influence on diabetes-related distress (β=−0.076, p<0.001).

## Discussion

Most patients (94.57%) with T2DM were suffering from diabetes-related distress. Our PAID score was higher than that of Norway (5.3), Poland (11) and Korea (8.82), but lower than in other studies conducted in Hunan province in China (43.06), southern India (27.5) and Greece (19.4).^43–48^

Table 3 shows that the type of medical alliance significantly influenced diabetes-related distress, with compact medical alliances reducing diabetes-related distress more than general medical alliances. Second, self-efficacy mediated the association of diabetes-related distress and the type of medical alliance. Previous studies also found that self-efficacy significantly mediated social support and diabetes distress;^31^ diabetes knowledge and diabetes self-management behaviour;^32^ diabetes distress and depressive symptoms, self-care behaviours, glycaemic control and health-related quality of life^33^ and diabetes distress and resilience.^34^ This suggests that diabetic patients’ mental health, diabetes self-management^49,50^ and overall well-being and quality of life can be improved by increasing the level of diabetes self-efficacy. ^20,21,51^ Health policymakers should develop intervention strategies focusing on increasing diabetic patients’ self-efficacy.

Drawing on the extant literature, we set out the supporting evidence of compact alliances’ advantages over general medical alliances.

Compact medical alliances typically exhibit a higher degree of integration, closer cooperative relationships and more extensive resource sharing among medical institutions, physicians, nurses and other healthcare personnel, which enabled them to provide more continuous, comprehensive and efficient medical services than general medical alliances.^37^ A pivotal component of this integration was the adoption of a unified health information system, which facilitated the sharing of electronic medical records and ensured the interconnectivity of medical information.^35^ Such systems are instrumental in coordinating efforts across the alliance, improving the continuity of care. Compact medical alliances were also characterized by robust cooperative frameworks, which included formal cooperation agreements, shared medical responsibilities and collaborative academic research.^52^ Finally, compact medical alliances structured resource sharing, where a proactive approach towards resource sharing encompassed human resources, medical equipment, pharmaceuticals and clinical data.^53^ This collective resource management not only better met patient needs, but also enhanced the efficiency of resource utilization, reduced healthcare costs and elevated the quality of medical care.

General medical alliances had lower degrees of service integration, weaker cooperative ties and restricted resource sharing.^53^ General medical alliances lack of close interaction and coordination among participating institutions leads to suboptimal resource utilization, discontinuity in patient care and overall inefficiency in service provision. Previous empirical research supports these observations. Chinese studies of patient-centred integrated care showed compact medical alliances promoted continuity in healthcare^54^ and that integrated nursing care enhances the management of complications and patient self-efficacy in cases of coronary heart disease.^55^ Compact medical alliances integrate diabetic patient health services compared with general medical alliances, such as medication guidance, follow-up blood glucose monitoring, tracking diabetes symptoms, health behaviour guidance and treatments. Diabetic patients accessing compact medical alliances were able to obtain more instrumental support to solve problems and receive feedback,^56^ which supported health management self-efficacy. This argument is consistent with our tests that showed self-efficacy was a mediator between the type of medical alliance and diabetes-related distress. For the treatment of diabetes and other chronic diseases, we recommend accelerating the transformation from general to compact medical alliances.

In our study, the confounding variables played a significant role in diabetes-related distress. Similar to previous studies,^24–26^ we found that lower education levels were significantly associated with diabetes-related distress, probably related to poorer diabetes cognition and diabetes prevention and treatment knowledge, leading to lower self-efficacy. Diabetes patients with lower education levels should be the key target for future intervention strategies. Income and health expenditures had no influence on self-efficacy in model 2, but medium and higher income was significantly associated with higher diabetes-related distress, which was heterogeneous with the results of a previous study.^23^ Higher socio-economic status developed country residents had a lower risk of T2DM and higher socio-economic status developing country residents were more likely to develop T2DM. One argument is that developing country high socio-economic residents are more likely to be overweight and diabetic because their income level is related to a poor diet and low physical activity.^57,58^ Lower health expenditures were significantly associated with higher diabetes-related distress, probably reflecting lower accessibility to healthcare. For patients with diabetes, moderate and high levels of medical spending allowed diabetes monitoring and treatment, reducing diabetes-related distress. Health insurance schemes subsidized diabetes sufferers to develop tailored diabetes healthcare plans.

Our study also found that <6 h of sleep per day was correlated with higher diabetes-related distress, while >8 h of sleep per day was a protective factor for diabetes-related distress. Previous research found that ≤5 h of sleep increased diabetes prevalence and impairment in glucose tolerance tests.^59^ A nested case–control study also found that both too short and long sleep time were risk factors of T2DM among rural Chinese residents.^60^ Short sleep duration raises the likelihood of serious insulin resistance and glycaemic control problems.^61^ Medical alliances should focus on informing and educating diabetes patients about regulated sleep time, especially avoiding sleeping <6 h per night, using sleep logs to promote the length and quality of sleep.^21^

Previous studies in Vietnam,^22^ Thailand,^26^ Greece^27^ and Brazil^62^ also found that participating in regular physical exercise was a protective factor of diabetes-related distress. Recommending people with diabetes mellitus regularly participate in physical exercise helps control physiological indicators, such as blood sugars, and reduce psychological problems related to diabetes-related distress. Medical alliances that integrate a range of health management professions should provide comprehensive plans coordinating sleep, diet and exercise. Our results also indicate that diabetes cognition impacted diabetes-related distress. A higher level of disease cognition can help patients form a correct attitude to face and deal with the disease, reducing their anxiety, depression and other negative psychological states. We support educational campaigns on managing and treating diabetes, which would attenuate diabetes-related distress.

### Strengths and limitations

There are three main strengths in this study. First, this was the first study of the impact of different types of medical alliances on diabetes-related distress in China. Second, self-efficacy was found to be a mediator variable between the type of medical alliance and diabetes-related distress. Finally, diabetes cognition and key behaviour variables, such as sleep time and physical exercise, were included in the analysis.

There are a number of limitations. First, the data are self-reported and physiological indicators were not included, but should be included in future studies. Second, cross-sectional data meant we were unable to infer causal relationships between the dependent and independent variables. Future studies should collect panel data. Third, we only assessed compact and general medical alliances. Future studies should look at alliance-specific features, such as telemedicine and shared electronic health records. Finally, the sample was collected from Jiangsu province with future studies collecting a national sample. Generalizing our results to other provinces and regions in China, or to other countries, should be undertaken cautiously.

## Conclusions

Medical alliances in China reduced diabetes-related distress, with compact medical alliances reducing diabetes-related distress more than general medical alliances. The relationship between medical alliances and diabetes-related stress was partially mediated by self-efficacy. Lower education level, higher income and lower health expenditures were risk factors for diabetes-related distress, while self-efficacy, higher diabetes cognition, longer sleep time and regular physical exercise were significant protective factors of diabetes-related distress. Accelerating the transformation of China's medical alliance system towards compact-type alliances can reduce diabetes-related distress and provide integrated healthcare tailored programs to educate diabetics on managing their disease, regulating their sleep and promoting an exercise regime.

## Data Availability

The data presented in this study are available upon request to the corresponding author.
